# Pterostilbene Exhibits Broad‐Spectrum Antiviral Activity by Targeting the Enterovirus Capsid, Inactivating Viral Particles, Blocking Viral Binding, and Protecting Mice From Lethal EV‐A71 Challenge

**DOI:** 10.1002/ptr.8496

**Published:** 2025-04-16

**Authors:** Kuan‐Ting Chuang, Siao‐Cian Pan, Bor‐Luen Chiang, Shih‐Hsun Chen, Min‐Hsiung Pan, Yu‐Li Chen, Cheng‐Sheng Lin, Chun‐Kai Pan, Jing‐Yi Lin, Yu‐Li Lin

**Affiliations:** ^1^ Department of Medical Research National Taiwan University Hospital Taipei Taiwan; ^2^ Department of Pediatrics National Taiwan University Hospital Taipei Taiwan; ^3^ Graduate Institute of Immunology College of Medicine Taipei Taiwan; ^4^ Institute of Biochemical Sciences National Taiwan University Taipei Taiwan; ^5^ Institute of Food Science and Technology National Taiwan University Taipei Taiwan; ^6^ Research Center for Chinese Herbal Medicine Graduate Institute of Health Industry Technology, College of Human Ecology, Chang Gung University of Science and Technology Taoyuan Taiwan; ^7^ Department of Clinical Laboratory Sciences and Medical Biotechnology College of Medicine, National Taiwan University Taipei Taiwan

**Keywords:** antiviral, enterovirus, hSCARB2 transgenic mouse, molecular docking, pterostilbene, resveratrol

## Abstract

Human enteroviruses (EVs) are a major public health issue worldwide owing to their potential to cause respiratory illnesses, hand‐foot‐and‐mouth disease, and severe neurological complications. Currently, no effective drugs or multivalent vaccines are available. Pterostilbene (Pte), a naturally occurring compound found in blueberries and other plants, is a type of stilbene with a similar structure to resveratrol. Pterostilbene exerts antioxidant, anti‐inflammatory, and anticancer properties. However, few studies have explored its antiviral activity. This study aimed to investigate the anti‐enteroviral effect and mechanisms of Pte against EV‐A71 and EV‐D68. Cytotoxicity and antiviral assays were performed to assess the safety of Pte to cells and its antiviral effects against enteroviruses. Viral attachment, inactivation assays, cellular receptor binding, western blotting, time‐of‐addition and time‐of‐removal assays, particle stability thermal release assay, and molecular docking were performed to elucidate the antiviral mechanisms of Pte. Additionally, we validated the antiviral effects of Pte using in vivo experiments. Among the stilbenes examined, Pte exerted a broad‐spectrum inhibitory effect on various enteroviruses, including EV‐A71, EV‐D68, and coxsackieviruses at 40 μM, without cytotoxicity. Mechanistically, Pte significantly inhibited enteroviral attachment, inactivated viral particles, blocked viral binding to its receptors, and increased virion stability. Molecular docking analysis revealed that Pte occupied a hydrophobic pocket in viral protein 1, indicating a strong binding affinity and acting as an efficient inhibitor. Notably, sequence alignment of multiple enteroviruses indicated that the Pte‐interacting residues in VP1 were highly conserved. In vivo studies demonstrated that oral administration of Pte significantly alleviated infection symptoms and reduced mortality in hSCARB2 transgenic mice. Pte possesses potential application as a broad‐efficacy antiviral drug against enteroviral infections.

## Graphical abstract

Pterostilbene (Pte) binds to the VP1 hydrophobic pocket of enteroviruses, stabilizing the viral capsid and preventing uncoating and RNA release. In vitro, Pte inactivates virions and impairs viral attachment, entry, and replication. In vivo, oral administration of Pte in hSCARB2‐transgenic mice reduces clinical symptoms and improves survival, supporting its therapeutic potential against enterovirus infection.

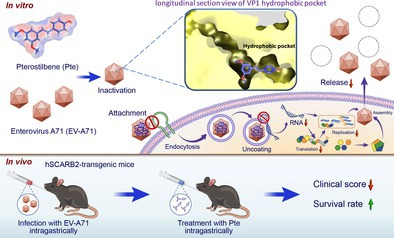

**Abbreviations**
AFM: acute flaccid myelitis
ATCC: American type culture collection
CC50: 50% cytotoxic concentration
CPE: cytopathic effect
CV‐A16: coxsackievirus A16
CV‐B3: coxsackievirus B3
DMEM: Dulbecco's modified Eagle's medium
dpi: days post‐infection
EC50: 50% effective concentrations
EV‐A71: enterovirus A71
EV‐D68: enterovirus D68
EVs: enteroviruses
FBS: fetal bovine serum
HED: human equivalent dose
hpi: hours post‐infection
MO/47: US/MO/14‐18947
MOI: multiplicity of infection
MP4: fourth passage of mouse‐adapted strain
MTT: 3‐(4,5‐dimethylthiazol‐2‐yl)‐2,5‐diphenyltetrazolium bromide
MWCO: molecular weight cut‐off
ORV: oxyresveratrol
Pic: piceatannol
Pin: pinostilbene
Pte: pterostilbene
RD cells: rhabdomyosarcoma cells
Res: resveratrol
TCID_50_
: 50% tissue culture infectious dose
VP1: viral protein 1

## 1 Introduction

Enteroviruses, positive‐sense single‐stranded RNA viruses that belong to the *Picornaviridae* family, cause various diseases and severe disorders, including respiratory illness, hand‐foot‐and‐mouth disease, viral meningitis, myocarditis, encephalitis, and acute flaccid myelitis (AFM) (Sun et al. 2019; Wang, Hu, et al. 2022). Enteroviruses are highly contagious, primarily spreading through the fecal‐oral route or respiratory droplets. Epidemic events associated with enteroviruses have been reported worldwide, posing a health risk to infants and children, with the main epidemic strains being EV‐A71, EV‐D68, and coxsackievirus (Souverbielle et al. 2023). Importantly, enteroviruses have an icosahedral capsid made up of four viral proteins (VP1, VP2, VP3, and VP4) that protect the viral RNA genome. VP1, the most external protein, plays a critical role in cell attachment. Upon infection, the virus binds to specific cellular receptors, which vary depending on the enterovirus species. Binding to these receptors induces a conformational change in the viral capsid, leading to the release of the viral RNA into the host cell, where it is directly translated, initiating the replication process. Understanding the interactions between enteroviruses and cellular receptors is crucial for developing targeted therapies and potential vaccines. Although licensed vaccines against the EV‐A71 are available in China (C4 sub‐genotype) and Taiwan (B4 sub‐genome), they only protect against EV‐A71 infection, without extending to EV‐D68 or coxsackievirus (Souverbielle et al. 2023; Wang, Hu, et al. 2022). Currently, no approved medication or multivalent vaccines against enteroviruses are available (Lin et al. 2023), and treatment is largely supportive. Hence, the development of antiviral drugs targeting multiple enteroviruses is urgently required.

Natural products have played pivotal roles in drug discovery and development, owing to their wide range of biological activities (Koh et al. 2024; Meng et al. 2019). Resveratrol (Res), pterostilbene (Pte), piceatannol (Pin), oxyresveratrol (ORV), and pinostilbene (Pin) are natural compounds belonging to the stilbene family and are characterized by their similar stilbene backbone (two aromatic rings linked by an ethene bridge). Among them, Res, the most well‐known compound, is found in grapes and berries and has been extensively studied for its antioxidant, anti‐inflammatory, anticancer, and cardiovascular protective properties (Koh et al. 2021). Pic is a metabolite of Res with an additional hydroxyl group, while ORV, commonly found in moraceous plants, exhibits greater metabolic stability and antioxidant potential than resveratrol (Likhitwitayawuid 2021). Both Pic and ORV have enhanced antioxidant activity owing to the presence of additional hydroxyl groups.

Pterostilbene, a dimethylated derivative of resveratrol, has been widely studied for its anti‐inflammatory, antioxidant, anticancer, and anti‐diabetes properties (Lin, Leland, et al. 2020). Notably, the lipophilic methoxy substitution in Pte leads to its improved metabolic stability, cellular uptake, and bioavailability compared with those of Res (Koh et al. 2021; Lin, Leland, et al. 2020). Pte undergoes demethylation to form its major metabolite, Pin.

A recent study demonstrated that Pte and Pin enhanced the innate immune response and restricted EV‐D68 infection (Yang et al. 2023). Res has been shown to inhibit EV‐A71 replication by blocking the IKKs/NF‐κB signaling pathway (Zhang et al. 2015). However, as these results were all from cell‐based experiments, the potential anti‐enteroviral activity of these stilbene compounds has not been extensively studied. The broad‐spectrum antiviral effects, mechanisms of action, and in vivo efficacy of pterostilbene against enteroviruses remain unexplored.

In this study, we evaluated the anti‐enteroviral activity of five stilbenes, namely Res, Pte, and their metabolites (ORV, Pic, and Pin). Additionally, we investigated the inhibitory effects and antiviral mechanisms of Pte on various enteroviral strains. Moreover, we validated the in vivo anti‐EV‐A71 efficacy of Pte using a hSCARB2 transgenic mouse model. Overall, this study provides novel insights into the antiviral mechanisms of Pte and highlights its potential as a therapeutic agent against enteroviruses.

## 2 Materials and Methods

### 2.1 Cells, Viruses, and Reagents

Human rhabdomyosarcoma (RD) cells (ATCC, Manassas, VA, USA) were cultured in Dulbecco's modified Eagle's medium (DMEM; SH30243.02; HyClone, Logan, UT, USA) supplemented with 10% fetal bovine serum (FBS; SH30396.03; HyClone) and antibiotic‐antimycotic solution, and maintained at 37°C in an incubator containing 5% CO_2_. The cells at passage 3–12 were used in this study. The EV‐A71 subclades used included C2 (TW/2272/98) and B4 (200307025). The EV‐D68 subclades included B3 (TW/5298/2016) and B1 (US/MO/14‐18947; MO/47). The coxsackievirus strains included A16 (CV‐A16; Tainan/5079/98) and B3 (CV‐B3). For the mouse experiments, the EV‐A71 TW/4643/MP4 strain (fourth passage of mouse‐adapted strain, genotype C2) with increased virulence in mice was used (Lin et al. 2021). Viruses were propagated in RD cells and stored at −150°C. Viral titers were determined using the Spearman–Kärber method and expressed as 50% tissue culture infectious dose (TCID_50_). Res, Pte, ORV, Piceatannol, and Pin (≥ 98.9%; MedChem Express) were prepared as stock solutions using dimethyl sulfoxide (DMSO), aliquoted, and maintained at −20°C. Catalogue numbers and lot numbers of all commercial antibodies are provided in Table S7.

### 2.2 Cytotoxicity Assay

The toxicity of stilbenes to RD cells was evaluated using the 3‐(4,5‐dimethylthiazol‐2‐yl)‐2,5‐diphenyltetrazolium bromide (MTT; Sigma, St Louis, MO, USA) assay. Cells (2 × 10^4^) were treated with compounds for 48 h. Subsequently, cells were replenished with 0.5 mg/mL of MTT for 1.5 h, and absorbance was measured at 570 nm using SpectraMax iD5 (Molecular Devices LLC., city, LA, USA). Cell viability was calculated using Equation (1):

(1) $$\text{Cell viability}\;\left(\%\right)=\frac{{\mathrm{OD}}_{\text{sample}}-{\mathrm{OD}}_{\mathrm{BK}}}{{\mathrm{OD}}_{\text{mock}}-{\mathrm{OD}}_{\mathrm{BK}}}\times 100\%$$

where OD is optical density, BK is blank, and mock is no drug treatment.

### 2.3 Antiviral Activity

Cells were infected with the virus at the indicated multiplicities of infection (MOI) for 1 h. After washing, infected cells were treated with stilbenes in a 2% FBS‐supplemented medium for 48 h. The antiviral activity was determined using the MTT assay and calculated according to Equation (2):

(2) $$\text{Antiviral activity}\;\left(\%\right)=\frac{{\left({\mathrm{OD}}_{\text{treat}}\right)}_{\text{virus}}-{\left({\mathrm{OD}}_{\text{ctrl}}\right)}_{\text{virus}}}{{\left({\mathrm{OD}}_{\text{ctrl}}\right)}_{\text{mock}}-{\left({\mathrm{OD}}_{\text{ctrl}}\right)}_{\text{virus}}}\times 100\%$$

where OD is optical density, mock is no viral infection, ctrl is no drug treatment, virus is viral infection, and treat is drug treatment.

Cell images were captured using an ImageXpress imaging system (Molecular Devices).

### 2.4 RNA Extraction and Real‐Time qPCR

Total RNA was extracted from RD cells and reverse‐transcribed into cDNA. Real‐time qPCR was performed using SYBR Green in a Bio‐Rad system (Bio‐Rad Laboratories, Hercules, CA, USA) with the following thermal cycling conditions: 95°C for 10 min, followed by 40 cycles of 95°C for 10 s and 60°C for 30 s. At the end of the amplification cycles, melting temperature analysis was performed. The expression levels were normalized to those of GAPDH. The primer sequences were as follows: EV‐A71 VP1: Forward (5′‐GCTTCTTCAGCAGAGCAGGATT‐3′) and reverse (5′‐GGGTTGGTTGTGCCTTCAAG‐3′); EV‐D68 VP1: Forward (5′‐AGCCTACCAGATCGAGAGCATC‐3′) and reverse (5′‐TTTCAACTGCATTTAAGCTAGGGAC‐3′).

### 2.5 Western Blotting

Cells were lysed using a RIPA lysis buffer for 30 min. Total protein was collected via centrifugation at 23,000 × g for 30 min at 4°C, quantified using the BCA assay, separated using SDS‐PAGE, and blotted onto PVDF membranes. After blocking with 5% bovine serum albumin (BSA), membranes were incubated overnight at 4°C with primary antibodies against EV‐A71 VP1 (MAB979; 1:2000, 38 kDa; MilliporeSigma, Burlington, MA, USA), EV‐D68 VP1 (GTX132313; 1:2000, 33 kDa; GeneTex, Irvine, CA, USA), and α‐tubulin (internal control; ab4074; 1:6000, 50 kDa; Abcam, Cambridge, UK). After incubation with the respective HRP‐conjugated secondary antibodies, target protein bands were visualized using ImageQuant LAS4000 (GE Healthcare, Chicago, IL, USA), and intensities were analyzed using ImageJ2 software.

### 2.6 Immunofluorescence Assay

After Pte treatment, infected cells were fixed in 4% paraformaldehyde and blocked with 3% BSA and 0.5% saponin for 30 min at 4°C. Thereafter, cells were treated with primary antibodies against VP1 for 40 min, followed by incubation for 30 min with secondary antibodies, including Alexa Fluor 488 anti‐mouse and Alexa Fluor 594 goat anti‐rabbit for detecting EV‐A71 and EV‐D68, respectively. Nuclei were visualized by performing Hoechst 33342 (0.5 μg/mL) staining for 10 min. Images were obtained using an ImageXpress imaging system and quantified using MetaXpress (Molecular Devices).

### 2.7 Time‐Of‐Addition Assay and Time‐Of‐Removal Assays

Briefly, RD cells (2 × 10^5^ cells/well) were seeded in 12‐well plates and incubated overnight, followed by infection with the virus at an MOI of 2.5 for 1 h. For the time‐of‐addition assay, the cells were treated with 20 μM of Pte at 0, 1, 3, and 5 h post‐infection (hpi). After one viral life cycle at 8 hpi, viral particles were collected from the culture supernatant and cells using thaw–freeze cycles, pooled, and the viral titer was evaluated using the TCID_50_ assay (Daelemans et al. 2011). For the time‐of‐removal assay, the cells were infected with the virus for 1 h, followed by Pte (20 μM) treatment at 0 hpi. Thereafter, the drug mixture was removed at 0, 1, 3, and 5 hpi by replacing it with fresh culture medium, followed by further incubation of the cells until 8 hpi. Finally, the viral particles were collected and processed as described above.

### 2.8 Viral Attachment Assay

Cells were co‐treated with the virus and compounds and incubated at 4°C for 1 h, which permitted the binding of viruses to the cell surface but restricted the internalization of viral particles into cells. After washing to remove unbound viruses, cells were harvested for RT‐qPCR.

### 2.9 Inactivation Assay

The virus was mixed with an equal volume of Pte and homogenized at 4°C for 1 h. Noninteracting compounds were removed from the mixture via centrifuging three times at 1500 × *g* for 10 min using a 50‐k molecular weight cut‐off (MWCO)‐Vivaspin filtration unit (Sartorius, Göttingen, Germany) (Hsieh et al. 2022). Thereafter, the collected viral particles were redispersed for titer determination using the TCID_50_ assay.

### 2.10 Pulldown Assay

Protein G Mag Sepharose Xtra magnetic beads (28‐9670‐70; Cytiva, Marlborough. MA, USA) were washed twice and incubated with 1 μg of recombinant human‐PSGL‐1‐Fc (3345‐PS; R&D Systems, Minneapolis, MO, USA), human‐SCARB2‐Fc (1966‐LM; R&D Systems), or human‐ICAM‐5‐Fc (1950‐M5; R&D Systems) protein for 1 h at 4°C. Viruses were mixed with DMSO or Pte in phenol red‐free medium for 1 h, followed by a MWCO filtration step. The mixtures were then incubated with the prewashed protein‐conjugated beads for 2 h. Next, the beads were washed thrice and mixed with sample buffer at 90°C for 10 min. Precipitated viruses and proteins were quantified via western blotting using anti‐EV‐A71 VP1, anti‐EV‐D68 VP1, mouse anti‐hPSGL‐1 (sc‐13535; 1:3000; Santa Cruz, Dallas, TX, USA), mouse anti‐hSCARB2 (sc‐55571; 1:2500; Santa Cruz), and mouse anti‐hICAM‐5 (1 μg/mL; MAB‐1950; R&D Systems) antibodies (Hsieh et al. 2022).

### 2.11 Particle Stability Thermal Release (PaSTRy) Assay

PaSTRy assay was performed to assess the stability of the viral particles, following a previously established method (Ho et al. 2016; Liu, Sheng, Fokine, et al. 2015). Briefly, 1 μg of EV‐A71 or EV‐D68 virion samples were prepared in a reaction mixture containing Pte (80 μM) or DMSO (control) in PBS and incubated at 37°C or 33°C for 1 h. SYBR Green II dye (Invitrogen) was added to each reaction at a final concentration of 6× according to the manufacturer's instructions, along with RNaseOUT (Invitrogen) at a concentration of 1 U/μL to prevent RNA degradation. A real‐time PCR system (Bio‐Rad CFX Opus 96) was utilized to generate a temperature gradient from 31°C to 90°C, with fluorescence readings taken at 1°C increments. At each temperature step, the mixture was heated for 2 min to ensure equilibrium and then cooled to 30°C for 2 min to stabilize the interaction of SYBR Green II with released RNA. This cooling step was necessary due to the temperature‐dependent fluorescence properties of SYBR Green II. The fluorescence intensity was plotted as a function of temperature, and the data were fitted with a sigmoidal curve.

### 2.12 Molecular Modelling

The capsid protein structures of EV‐A71 (3ZFF) and EV‐D68 MO/47 (7TAF) strains were obtained from the Protein Data Bank. The structures of the proteins and pterostilbene were prepared using BIOVIA Discovery Studio 2024 (Dassault Systems, San Diego, CA, USA). The protein‐deficient structure was repaired, and the ligand conformation was optimized for energy minimization. Initially, the binding site of the protein was determined based on the in situ compound coordinates in the crystal structure, followed by the refinement of the binding site. The docking analysis of Pte in the VP1 structure was performed using the CHARMm‐based CDOCKER. The parameters random conformation and orientations to refine were both set to 20. For each VP1 structure, the top 10 conformations with the best CDOCKER scores were retained. The binding free energy was predicted using the MM‐PBSA program with the Poisson‐Boltzmann with nonpolar surface area implicit model. Hydrophobic and hydrogen bonding interactions between VP1 and Pte were visualized in 2D diagrams.

### 2.13 Mouse Protection Assay

All animal experiments were approved by the Institutional Animal Care and Use Committee (Approval No: 20201172) of the College of Medicine, National Taiwan University (Taipei, Taiwan), and performed in accordance with the approved guidelines.

Briefly, hSCARB2 transgenic mice (21‐d‐old) obtained from the National Laboratory Animal Center (Taipei, Taiwan) were randomly assigned to experimental groups, with no significant difference in the average initial body weight of each group. Mice were infected with EV‐A71/MP4 (1 × 10^7^ PFU/mouse) intra‐gastrically (i.g.). After 1 h, mice were i.g. treated with PBS or Pte (20 and 50 mg/kg). Mice in all groups were treated once daily for a total of 7 days and monitored daily for pathological signs. Experiments were not blinded. Clinical scores were assigned as previously described (Lin, Shih, et al. 2020). The body weight and survival rates of mice were observed daily for 14 days after injection.

### 2.14 Statistical Analysis

All data analyses were performed using GraphPad Prism 9 software (La Jolla, CA, USA). Experimental data are presented as mean ± standard error of the mean (SEM). Statistical comparisons between two groups were performed using the Student's *t*‐test, whereas multi‐group comparisons were conducted using one‐way or two‐way analysis of variance (anova). All experiments were performed in triplicate. Statistical significance was set at *p* < 0.05.

## 3 Results

### 3.1 Anti‐Enteroviral Activities of Stilbene Analogues

**FIGURE 1:**
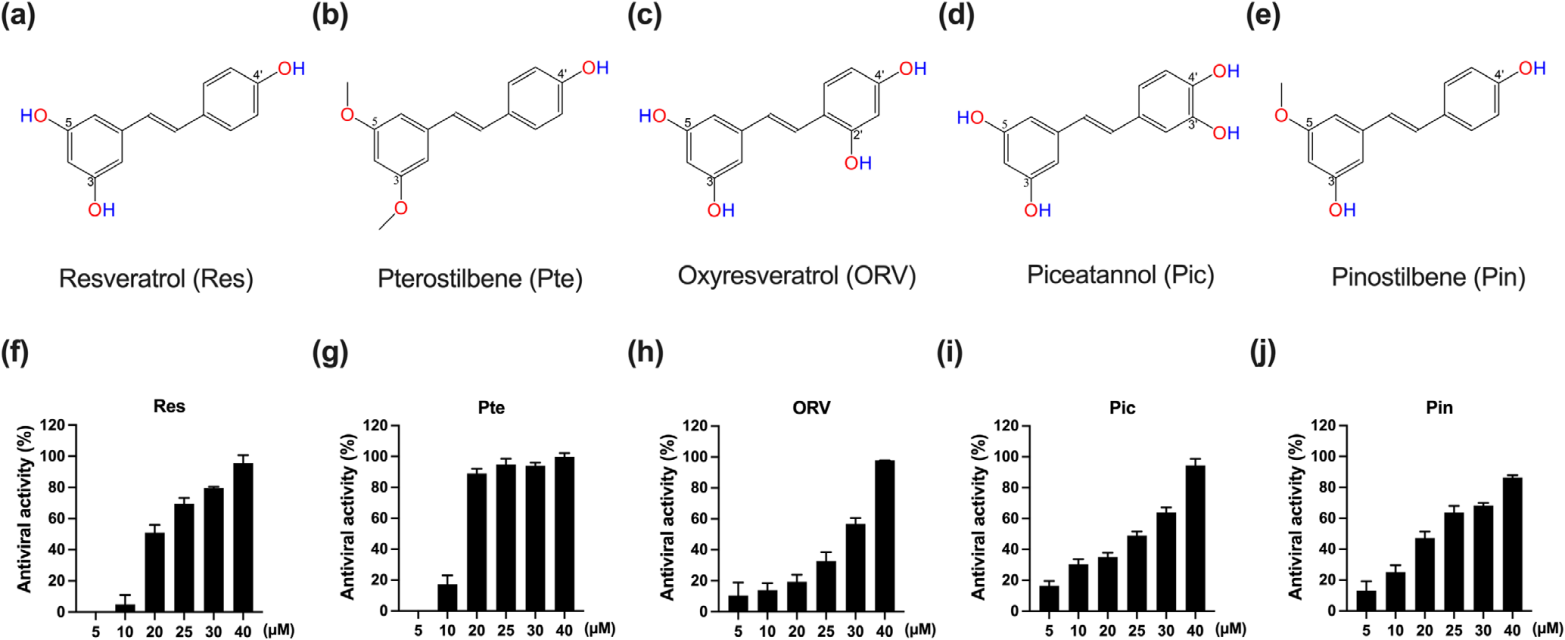
Antiviral effects of stilbene analogues. Chemical structure of (a) resveratrol (Res), (b) pterostilbene (Pte), (c) oxyresveratrol (ORV), (d) piceatannol (Pic), and (e) pinostilbene (Pin). The antiviral activity of (f) Res, (g) Pte, (h) ORV, (i) Pic, and (J) Pin against EV‐D68 infection in RD cells. Cells were infected with 0.1 MOI EV‐D68 B3 for 1 h; after washing, cells were treated with increasing concentrations of stilbenes for 48 h. Cell viability was determined using the MTT assay. The antiviral activities of these stilbenes were calculated according to Equation (2) described in Section 2. *N* = 4. Data are expressed as the mean ± SEM.

We investigated the anti‐enteroviral activities of five common stilbene analogues. The structure of these stilbenes (Res, Pte, ORV, Pic, and Pin) is shown in Figure 1a–e. To determine the antiviral activities of these stilbenes against EV‐D68 infection, RD cells were infected with EV‐D68 and then treated with Res, Pte, ORV, Pic, and Pin at different concentrations. We evaluated the antiviral activities of the five stilbenes using MTT assays. We found that the anti‐EV‐D68 activities of the five stilbenes increased in a concentration‐dependent manner (from 5 to 40 μM; Figure 1f–j). The MTT test showed that the 50% effective concentrations (EC_50_) of Res, Pte, ORV, Pic, and Pin were 19.6, 12.7, 27.9, 25.4, and 20.4 μM, respectively. These results indicated that the antiviral efficacy of the five stilbenes against EV‐D68 manifested in the following order: Pte > Res > Pin > Pic > ORV. Therefore, Pte and Res were selected for further investigation of their antiviral activities against other enteroviral strains.

### 3.2 Pte and Res Exhibited Broad‐Spectrum Antiviral Effects Against Multiple Enteroviral Strains

**FIGURE 2:**
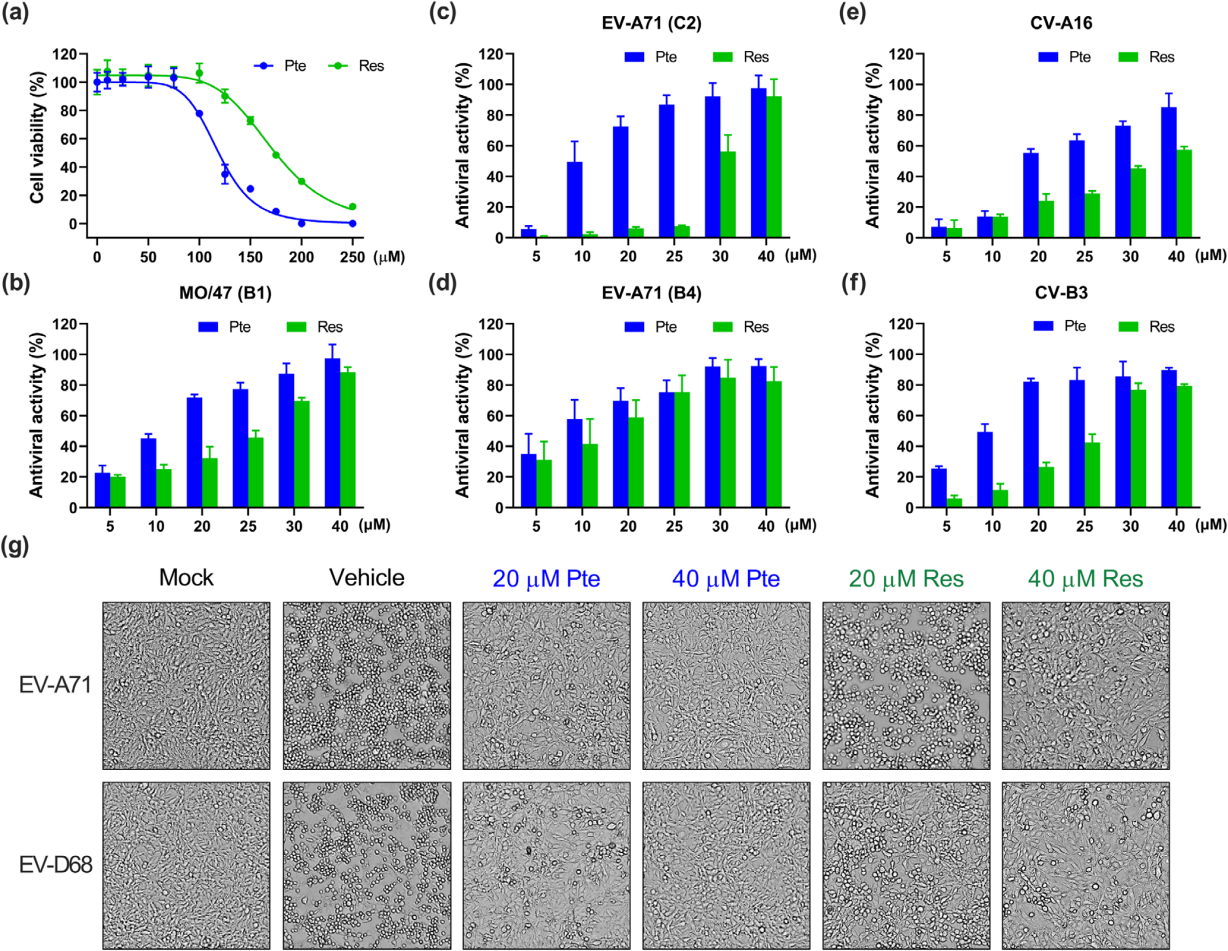
Antiviral effects of pterostilbene and resveratrol. (a) Cytotoxicity of Pte and Res in RD cells. Cells were seeded in 96‐well plates and treated with increasing concentrations of Pte and Res for 48 h. Cell viability was determined using the MTT assay (*N* = 5). Antiviral activity of Pte and Res against (b) EV‐D68 MO/47 B1, (c) EV‐A71 C2, (d) EV‐A71 B4, (e) CV‐A16, and (f) CV‐B3 (MOI = 0.1) infection. RD cells were infected with enteroviruses and then treated with various concentrations of Pte or Res as indicated. The effects of Pte and Res on cell viability was determined using MTT assays. (g) Cell morphology of EV‐A71‐ and EV‐D68‐infected cells treated with Pte or Res (20 and 40 μM) under a phase‐contrast microscope (Olympus) (100× magnification). The mock group was treated with PBS, whereas the vehicle group was infected but not treated. *N* = 4. All experiments were performed at least in quadruplicate. Data are expressed as the mean ± SEM.

Based on the antiviral effect of Pte and Res against EV‐D68, we further investigated the broad‐spectrum efficacy of Pte and Res against other enteroviruses. First, we confirmed that treatment with Pte and Res at concentrations below 75 μM had no indirect or direct cytotoxic effect on cell proliferation (Figure 2a), with 50% cytotoxic concentration (CC_50_) of 119 μM for Pte and 168 μM for Res. To detect the antiviral activities of Pte and Res against various enteroviruses, RD cells were infected with EV‐D68 (B3), EV‐D68 (MO/47; B1), EV‐A71 (C2), EV‐A71 (B4), CV‐A16, and CV‐B3. After viral adsorption for 1 h, the medium was removed, and the cells were treated with various concentrations of Pte and Res, followed by determination of cell viability using MTT assays (Figure 2b–f). The EC_50_ values of Pte against EV‐D68 (B3), EV‐D68 (MO/47; B1), EV‐A71 (C2), EV‐A71 (B4), CV‐A16, and CV‐B3 were 12.7, 10.9, 11.3, 8.2, 18.7, and 11.0 μM, respectively, whereas those of Res were 19.6, 27.5, 29.6, 18.0, 35.6, and 6.6 μM, respectively (Table 1). Additionally, we further investigated the antiviral activity of Pte and Res by performing an anti‐cytopathic effect (CPE) assay. Pte treatment was significantly better than Res in protecting RD cells from EV‐A71 and EV‐D68 infection, whereas untreated control cells (vehicle) infected with EV‐A71 and EV‐D68 acquired rounded morphology due to CPE (Figure 2g). Taken together, these results demonstrated that Pte has a superior anti‐enteroviral effects compared to Res. Therefore, Pte was selected for further investigation.

**TABLE 1:** Antiviral effects of pterostilbene (Pte) and resveratrol (Res) against different enteroviral strains.

	Compounds	CC_50_ (μM)	EC_50_ (μM)	SI
RD Cells	Pte	118.9 ± 2.8		
	Res	167.9 ± 6.4		
EV‐A71 C2	Pte		11.3 ± 2.3	10.5
	Res		29.6 ± 1.2	5.7
EV‐A71 B4	Pte		8.2 ± 3.1	14.5
	Res		18.0 ± 5.2	9.3
EV‐D68 5298 B3	Pte		12.7 ± 1.4	9.4
	Res		19.6 ± 3.9	8.6
EV‐D68 MO/47 B1	Pte		10.9 ± 1.8	10.9
	Res		27.5 ± 2.3	6.1
CV‐A16	Pte		18.7 ± 4.1	6.4
	Res		35.6 ± 3.2	4.7
CV‐B3	Pte		11.0 ± 1.7	10.8
	Res		25.5 ± 1.6	6.6

*Note*: CC_50_: 50% cytotoxic concentration of the drug; EC_50_: drug concentration that inhibits 50% virus‐induced cytopathic effect; SI: selectivity index, defined as CC_50_/EC_50_. *N* = 4. Data are expressed as the mean ± SEM.

### 3.3 Pte Inhibited Progeny Virus, Viral RNA Replication, and VP1 Production

**FIGURE 3:**
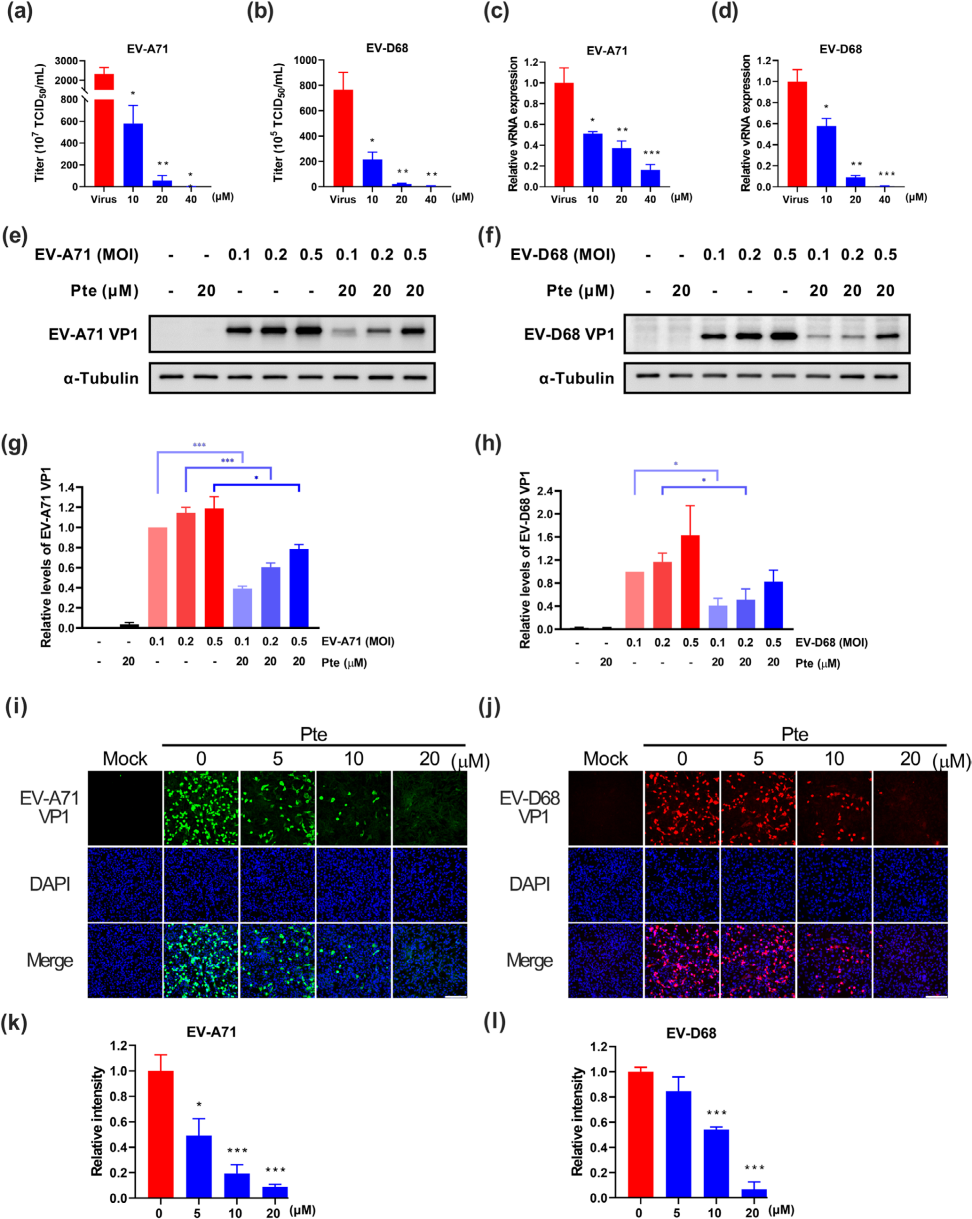
Pterostilbene inhibits viral progeny production, RNA expression, and VP1 protein production. The effect of Pte on infectious viral progeny production was investigated. RD cells were infected with (a) EV‐A71 C2 or (b) EV‐D68 B3 (0.1 MOI) for 1 h, and subsequently treated or not with Pte for 48 h. The culture supernatants were collected and the viral titers were determined using the TCID_50_ assay (*N* = 3). RD cells were infected with (c) EV‐A71 or (d) EV‐D68 at an MOI of 0.1 for 1 h, and then cells were treated with indicated concentrations of Pte for 24 h. The levels of viral RNA were quantified using RT‐PCR, with the expression of the VP1 gene being normalized to that of GAPDH (*N* = 3). The protein levels of VP1 were detected using western blot analysis. RD cells were infected with (e) EV‐A71 or (f) EV‐D68 at MOI of 0.1, 0.2, and 0.5 for 1 h, and then cells were treated with 20 μM Pte for 24 h. Densitometry of (g) EV‐A71 VP1 and (h) EV‐D68 VP1 western blot (*N* = 4). RD cells were mock‐infected or infected with (i) EV‐A71 or (j) EV‐D68 at a MOI of 0.1 for 1 h, and then cells were treated with Pte for 24 h. The amount of viral particles were assessed using immunofluorescence staining and subsequent image analysis. Mock infection was used as a control. Immunofluorescence signals of (k) EV‐A71 and (l) EV‐D68 were quantified as VP1 fluorescence relative to the nuclear signal, with background subtraction, and expressed as a ratio to that of the virus control group (*N* = 6). Scale bar = 200 μm. Data are expressed as the mean ± SEM.*: *p* < 0.05, **: *p* < 0.01, ***: *p* < 0.001 versus the virus control group.

To verify whether Pte inhibited viral replication, we monitored the viral titer, RNA expression, and protein synthesis in infected RD cells. The viral titers of EV‐A71 and EV‐D68 after Pte treatment were significantly lower than those in untreated control cells (virus) (*p* < 0.01) in a dose‐dependent manner. Specifically, treatment with Pte at concentrations ≥ 20 μM caused more than a 33‐fold decrease in viral titer (Figure 3a,b). Furthermore, we found that Pte treatment mediated a significant dose‐dependent decrease in the viral RNA levels of EV‐A71 and EV‐D68 at 24 hpi (Figure 3c,d). To investigate the effect of Pte on viral protein expression, RD cells were infected with EV‐A71 and EV‐D68 at 0.1, 0.2, or 0.5 MOI for 1 h, then treated with or without Pte. We observed that Pte treatment downregulated the levels of VP1 protein by approximately 32%–61% in EV‐A71‐infected cells (Figure 3e,g) and 45%–60% in EV‐D68‐infected cells (Figure 3e–h) at different MOIs. Immunofluorescence staining showed reduced synthesis of VP1 protein for both EV‐A71and EV‐D68 in Pte‐treated cells (Figure 3i,j). Analysis of integrated signals revealed that Pte treatment significantly reduced VP1 protein levels in a dose‐dependent manner (Figure 3k,l).

### 3.4 Pte Especially Affected the Early Stage of Viral Replication

**FIGURE 4:**
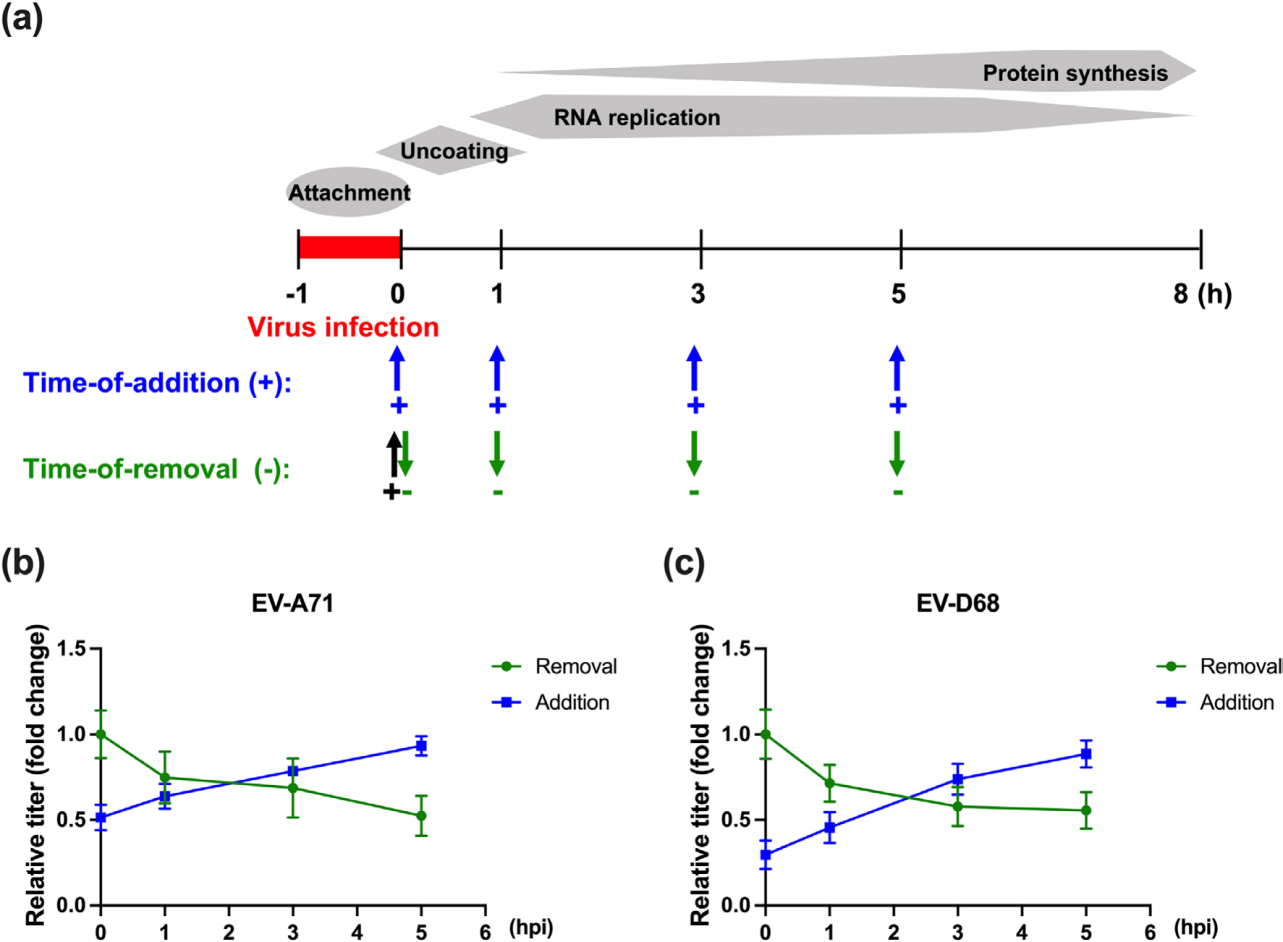
Time‐of‐addition and time‐of‐removal assays of pterostilbene in cells infected with EV‐A71 or EV‐D68. (a) Schematic layout of time‐of‐addition and time‐of‐removal assay with various stages of the virus life cycle. Cells were seeded in 12‐well plates and infected with (b) EV‐A71 or (c) EV‐D68 for 1 h. Following washing, for the time‐of‐addition assay, cells were treated with 20 μM of Pte at 0, 1, 3, and 5 hpi. For the time‐of‐removal assay, Pte was added at 0 hpi and subsequently removed at 0, 1, 3, and 5 hpi. At 8 hpi, the culture supernatants and cells were collected, and the viral titers were determined using the TCID_50_ assay. *N* = 4. Data are expressed as the mean ± SEM.

To investigate the stage(s) of the enterovirus replication cycle affected by Pte, time‐of‐addition and time‐of‐removal assays were performed using a single‐cycle infection format. As illustrated in the schematic diagram (Figure 4a), Pte (20 μM) was added at various time points post‐infection for the time‐of‐addition assay. For the time‐of‐removal assay, Pte was added immediately after infection and subsequently removed at different time points. Viral titers relative to untreated infected cells (virus control) were measured at 8 hpi. The addition of Pte at 0 and 1 hpi significantly reduced the viral titers of EV‐A71 and EV‐D68 (Figure 4b,c). Introducing Pte at later time points (3 and 5 hpi) resulted in a progressive increase in viral titers, indicating that its antiviral efficacy was strongest when administered early. Time‐of‐removal experiments revealed that removing Pte at 1 hpi did not significantly inhibit viral replication, whereas removal at 3 and 5 hpi significantly reduced viral titers (Figure 4b,c). Overall, these results suggested that Pte exerts its antiviral activity during the early stages of EV‐A71 and EV‐D68 infection.

### 3.5 Pte Interfered With Enteroviral Attachment, Activation, Binding to Specific Receptors, and Increasing the Virion Stability

**FIGURE 5:**
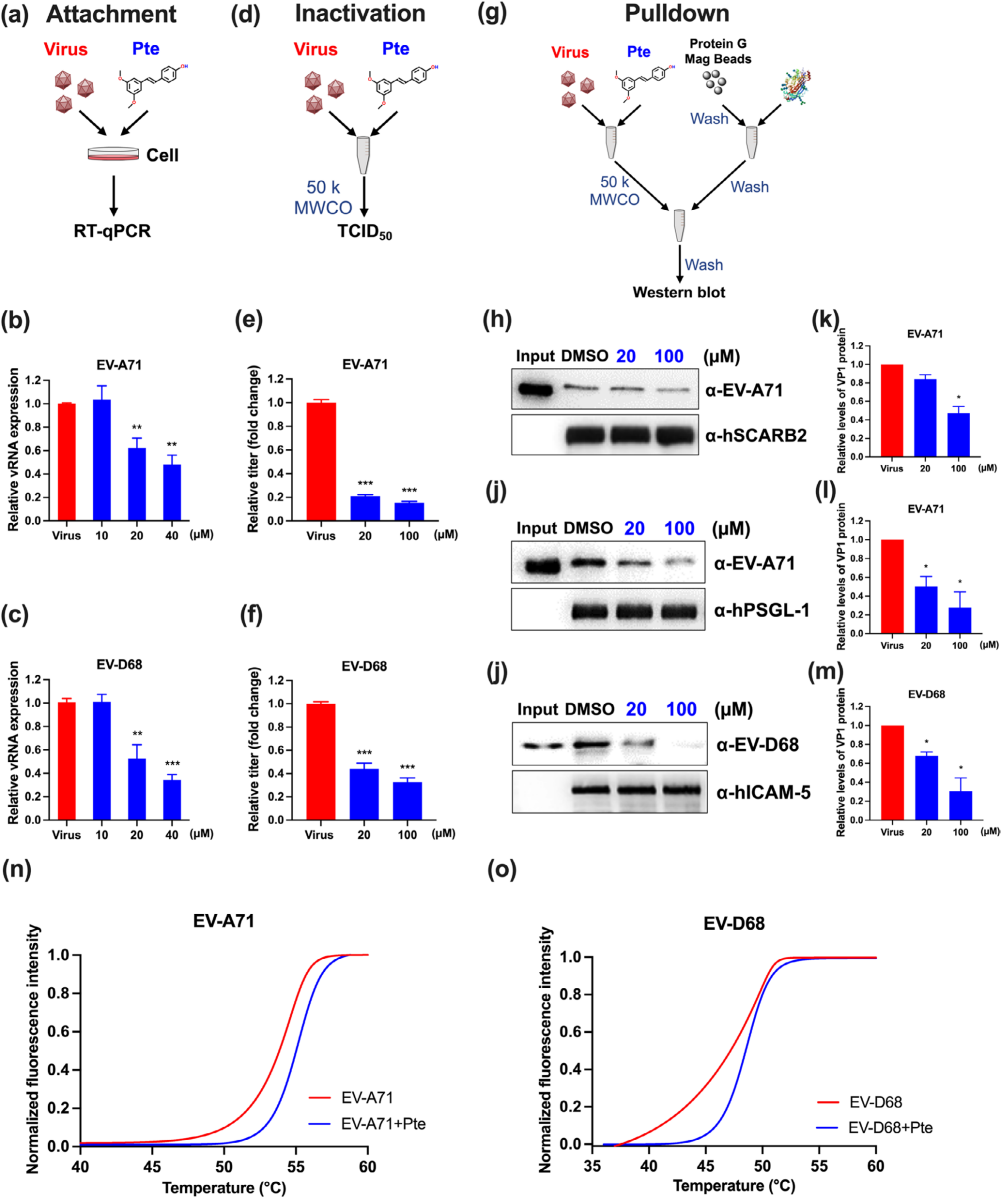
Effects and mechanisms of action of pterostilbene. (a) Schematic diagram of the attachment assay. Cells were incubated with drug‐virus mixtures at 4°C for 1 h, followed by cell harvesting for detecting viral RNA expression using RT‐qPCR. The interference effects of Pte on the attachment of (b) EV‐A71 and (c) EV‐D68 at an MOI of 5 are presented as the relative expression level compared with that of the virus control group. (d) Schematic diagram of the inactivation assay. The compound‐virus mixture was homogenized at 4°C for 1 h, followed by centrifugal filtration using a 50 kDa‐MWCO unit. The inactivation effects of Pte against (e) EV‐A71 and (f) EV‐D68 are expressed as a ratio relative to that of the virus control group at a final titer of 1 × 10^5^ PFU/mL, as determined using TCID_50_. (g) Experimental procedure of the receptor pulldown assays. Viruses (TCID_50_ = 10^8^) were preincubated with Pte or DMSO, and then subjected to MWCO filtration before binding to receptor protein‐conjugated beads; 1 μg of virus served as an input control. The expression of VP1 with precipitated (h) hSCARB2, (i) hPSGL‐1, and (j) hICAM‐5 was determined using western blotting. Densitometry of EV‐A71 or EV‐D68 VP1 protein level related to (k) hSCARB2, (l) hPSGL‐1, or (m) hICAM‐5 western blot, respectively. The virion stability. SYBR Green II fluorescent assay to measure stability of EV‐A71 and EV‐D68 particles. (n) EV‐A71 and (o) EV‐D68 virions were mixed with SYBR Green II fluorescent dye and heated to indicated temperatures. The fluorescent signal increases as the dye binds to RNA that is release from thermally destabilized particles. *N* = 3. Data are expressed as the mean ± SEM.*: *p* < 0.05, **: *p* < 0.01, ***: *p* < 0.001 versus the virus control group.

To gain further mechanistic insights, we performed attachment, centrifugal filtration inactivation, and receptor pulldown assays. We performed viral attachment experiments to determine whether the anti‐enteroviral activity of Pte was related to inhibiting viral adhesion and preventing the entry of viruses into cells. RD cells were prechilled at 4°C and then infected with EV‐A71 or EV‐D68 in the presence or absence of Pte on ice for 1 h (Figure 5a). We found that treatment with 20 and 40 μM of Pte significantly reduced viral titers (Figure 5b,c), indicating that Pte inhibited viral attachment. We further investigated whether Pte could directly target viral particles or interfere with the binding of viruses to specific receptors. To this aim, EV‐A71 or EV‐D68 was preincubated with Pte. After removal of Pte via filtration, the titer of EV‐A71 or EV‐D68 was quantified (Figure 5d). We found that both EV‐A71 and EV‐D68 titers were significantly reduced in a dose‐dependent manner (*p* < 0.001). Compared with the untreated control (virus), Pte‐treated virus showed an approximately 80% and 60% reduction in EV‐A71 and EV‐D68 titers, respectively (Figure 5e,f), indicating that Pte directly targeted EV‐A71 and EV‐D68 particles. These results indicated that Pte not only affected the ability of the virus to bind to cells but also directly targeted viruses. We next investigated whether Pte interferes with the interaction between the virus and host receptor during viral infection. We performed receptor pulldown assays to detect interactions between the virus and known EV‐A71 receptors, such as hSCARB2 and hPSGL‐1, or EV‐D68 receptors, such as hICAM‐5, in the presence of Pte. EV‐A71 or EV‐D68 was preincubated with Pte or DMSO before being precipitated with hSCARB2 and hPSGL‐1, or hICAM‐5 (Figure 5g). We observed that Pte effectively inhibited the binding of both EV‐A71 and EV‐D68 to their specific receptors in a dose‐dependent manner, as detected by western blotting using anti‐EV‐A71 (Figure 5h,i) or anti‐EV‐D68 antibodies (Figure 5j). Furthermore, a modified PaSTRy assay was performed to monitor viral uncoating, during which the closed viral protein shell undergoes reconfiguration to release viral RNA or become permeable to fluorescent dyes. Compared with that in the control (DMSO) group, a temperature increase of 2°C was required to release the RNA genome when EV‐A71 and EV‐D68 were incubated with 80 μM of Pte (Figure 5n,o), indicating that Pte improved the stability of EV‐A71 and EV‐D68 capsids and prevented the virus from uncoating during viral entry. Together, these results suggested that Pte directly targeted EV‐A71 or EV‐D68, and thus interfered with the EV‐A71‐hSCARB2, EV‐A71‐hPSGL‐1, or EV‐D68‐hICAM‐5 interaction.

### 3.6 Molecular Docking Analysis to Simulate Pte and VP1 Interaction

**FIGURE 6:**
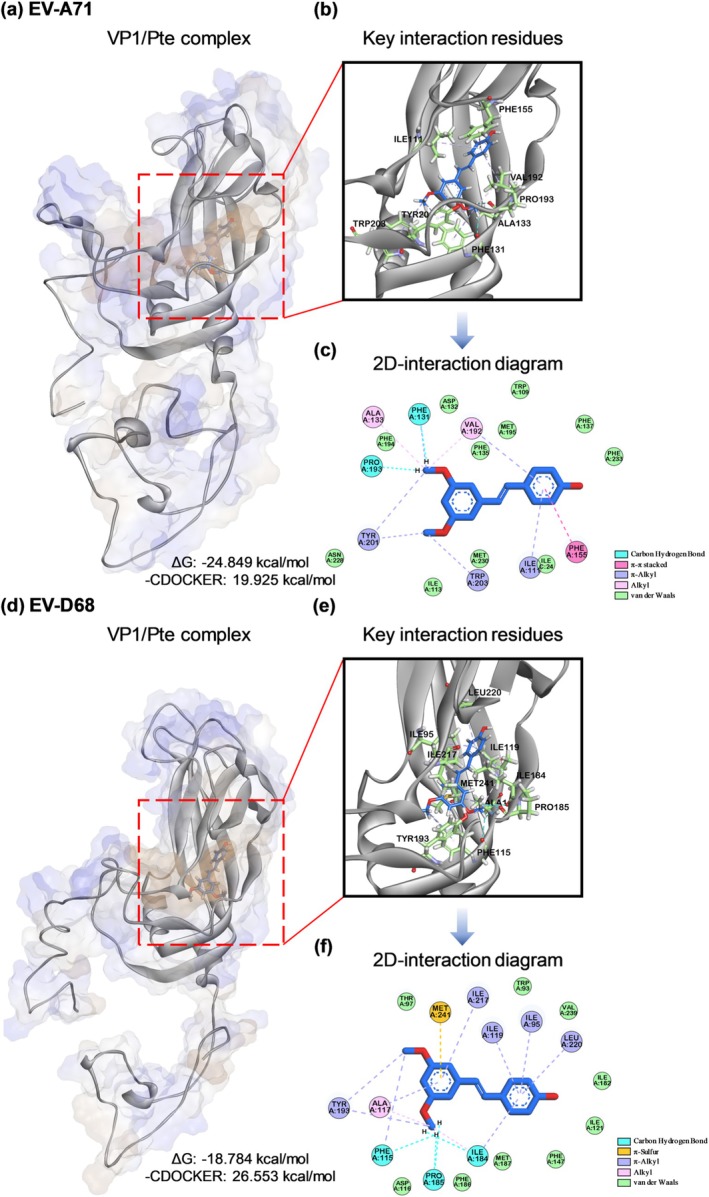
Docking pose analysis of pterostilbene in the VP1 hydrophobic pockets of EV‐A71 and EV‐D68. The docking sites of Pte was simulated using the VP1 hydrophobic pocket of (a) EV‐A71 (3ZFF) and (d) EV‐D68 (7TAF). Pte is shown as blue stick structures. The corresponding binding free energies (ΔG) and ‐CDOCKER energies are described in units of kcal/mol. VP1 is depicted as a grey cartoon with a hydrophobic surface (blue‐white‐brown gradient). Hydrophobic pockets are presented as brown surfaces. (b) and (e) In the key interactive residues, the labelled interacting residues on VP1 are shown as green stick structures. (c) and (f) In the 2D‐interaction diagram, the hydrophobic π‐alkyl, alkyl, π‐π stacked, and π‐sulfur interactions are drawn as pink, purple, hotpink, and yellow dashed lines, respectively. Hydrogen bonds are presented in cyan. while van der Waal forces are shown in green. VP1 is shown in cartoon representation in blue with side chains of residues forming the hydrophobic pocket shown as sticks.

In silico molecular modeling was performed to explore the docking pose and interaction forces between Pte and VP1 residues of EV‐A71 or EV‐D68. We first identified that Pte was anchored securely within the pocket of VP1 (Figure 6). These pockets serve as potent inhibitory sites in EV‐A71 and EV‐D68 strains (Liu, Sheng, Fokine, et al. 2015; Wang, Hu, et al. 2022). Notably, the pocket space in EV‐D68 is distinctly shallower and narrower than that in EV‐A71, possibly insufficient for binding to immunoglobulin‐like receptors but permitting sialylated receptor binding (Liu, Sheng, Baggen, et al. 2015; Liu, Sheng, Fokine, et al. 2015). Next, we observed that Pte generated stable noncovalent interactions in the hydrophobic pocket. Pte interacted with EV‐A71 VP1 by forming six π‐alkyl hydrophobic interactions with Ile111, Phe131, Val192, Tyr201, and Trp203, and two alkyl hydrophobic interactions with Ala133 and Val192. Additionally, we detected a π‐π stacking interaction with Phe155 and hydrogen bonds with Phe131 and Pro193. Other surrounding residues contributed to van der Waals forces (Figure 6c). Whereas, Pte interacted with EV‐D68 VP1 by forming nine π‐alkyl hydrophobic interactions with Ile95, Phe115, Ala117, Ile119, Ile184, Tyr193, Ile217, and Leu220, and two alkyl hydrophobic interactions with Ala117 and Ile184. Moreover, we identified a π‐sulfur interaction with Met241 and hydrogen bonds with Phe115, Ile184, and Pro185. Other surrounding residues also contributed to van der Waals forces (Figure 6f). Importantly, the binding free energy of Pte in EV‐A71 and EV‐D68 was predicted to be −24.849 and −18.784 kcal/mol, respectively, and ‐CDOCKER energies were 19.925 and 26.553 kcal/mol, respectively.

### 3.7 Conservation of Pte‐Binding Sites Across VP1 Proteins of Enterovirus

To determine the similarity of the hydrophobic pocket binding sites of Pte across the tested enteroviruses. We analyzed the molecular docking of Pte and the reference inhibitors within the VP1 hydrophobic pocket of the four enteroviruses (Figure S1). The results of the molecular docking studies revealed that Pte mostly interacted with the hydrophobic side chains of the residues forming the pocket interior. Pte binds to the VP1 hydrophobic pocket of EV‐A71, EV‐D68, CV‐A16, and CV‐B3; these key interacting residues are also involved in the binding of known capsid‐inhibitors, such as WIN51711, pleconaril, and GPP3 (De Colibus et al. 2015; Muckelbauer et al. 1995; Plevka et al. 2013) (Figure S2). The results of structural comparisons indicated a high degree of similarity in the binding residues of Pte across these enteroviruses, as shown in the 2D interaction maps. In Table S1, the colored amino acids highlight the interacting residues that are identical within the hydrophobic pocket of Pte and known VP1‐target inhibitors, indicating similar binding properties. The docking results suggested that Pte stabilizes the viral capsid similarly to these established antiviral compounds, preventing uncoating and viral RNA release.

**FIGURE 7:**
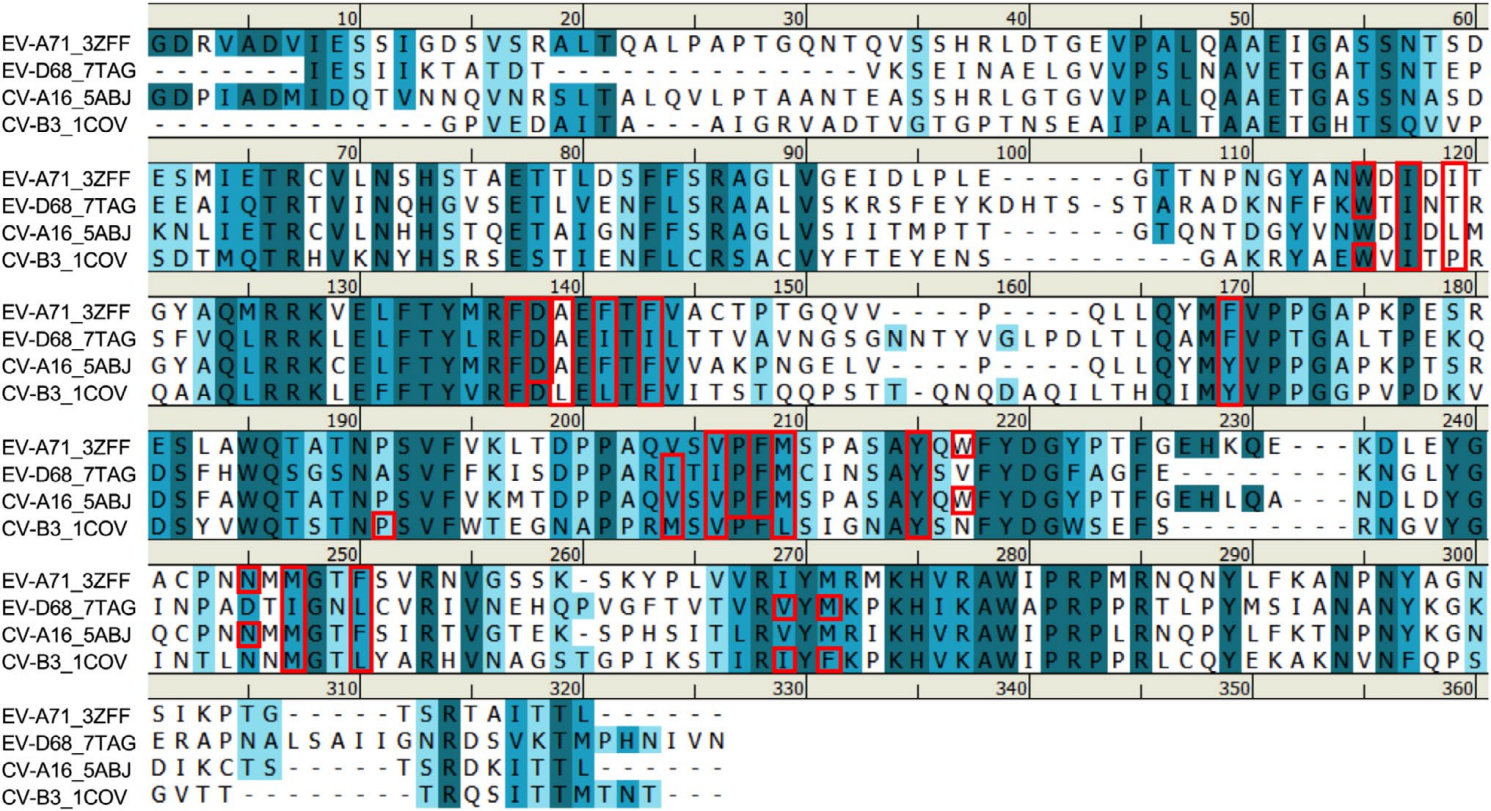
Multiple sequence alignment of VP1 capsid proteins from EV‐A71 (PDB: 3ZFF), EV‐D68 (PDB: 7TAG), CV‐A16 (PDB: 5ABJ), and CV‐B3 (PDB: 1COV). The numbering of amino acids was according to EV‐A71 primary amino acid sequence. Dark blue amino acids indicate complete conservation, medium blue residues represent strong similarity, and light blue residues show moderate similarity in chemical properties. Pterostilbene‐binding sites within the VP1 hydrophobic pocket were marked with red boxes. This figure was generated using BIOVIA Discovery Studio 2024 (Dassault Systems, San Diego, CA, USA).

In addition, we assessed the multiple sequence alignment of VP1 capsid proteins from four enteroviruses (EV‐A71, EV‐D68, CV‐A16, and CV‐B3) to compare the conservation of Pte binding sites among these viruses (Figure 7). Pte binding sites within the VP1 hydrophobic pocket are marked with red boxes in Figure 7. The results showed that several key residues within the binding pocket are highly conserved across all four enteroviruses. This suggests that Pte may exert broad‐spectrum antiviral effects by targeting the conserved structural elements within VP1 and stabilizing the virion. Additionally, we extended our sequence analysis to multiple genotypes of each enterovirus. In EV‐A71, sequence alignment across 14 genotypes (A, B0–B5, and C1–C5) showed that all Pte‐binding sites were completely conserved (Table S2). Similarly, EV‐D68 sequence alignment (MO/14‐18947, KY/14‐18953, TW‐3416‐2016, and Fermon) exhibited minimal variation within Pte‐binding residues (Table S3). The only observed substitution (Met at position 182) involved chemically similar residues (Ile → Met), suggesting that Pte‐interacting residues remain highly conserved. Most of the Pte‐interacting residues are highly conserved among the different CV‐A genotypes (Table S4). Minor amino acid variations, such as substitutions at positions 113, 155, 192, 195, and 230, were observed; however, the chemical properties at these positions are highly similar. The VP1 binding sites for Pte in CV‐B are relatively highly conserved across the genotypes (B1–B6) (Table S5). Some variations were observed at positions 92, 114, 180, 183, and 216; however, the chemical properties at these positions are highly similar. These data demonstrated that the residues interacting with Pte are highly conserved despite minor residues variations, supporting the broad‐spectrum efficacy of Pte against multiple enterovirus strains, which was confirmed in an inhibition spectrum assay (Table 1).

### 3.8 In Vivo Efficacy of Pte in EV‐A71‐Infected Mice

**FIGURE 8:**
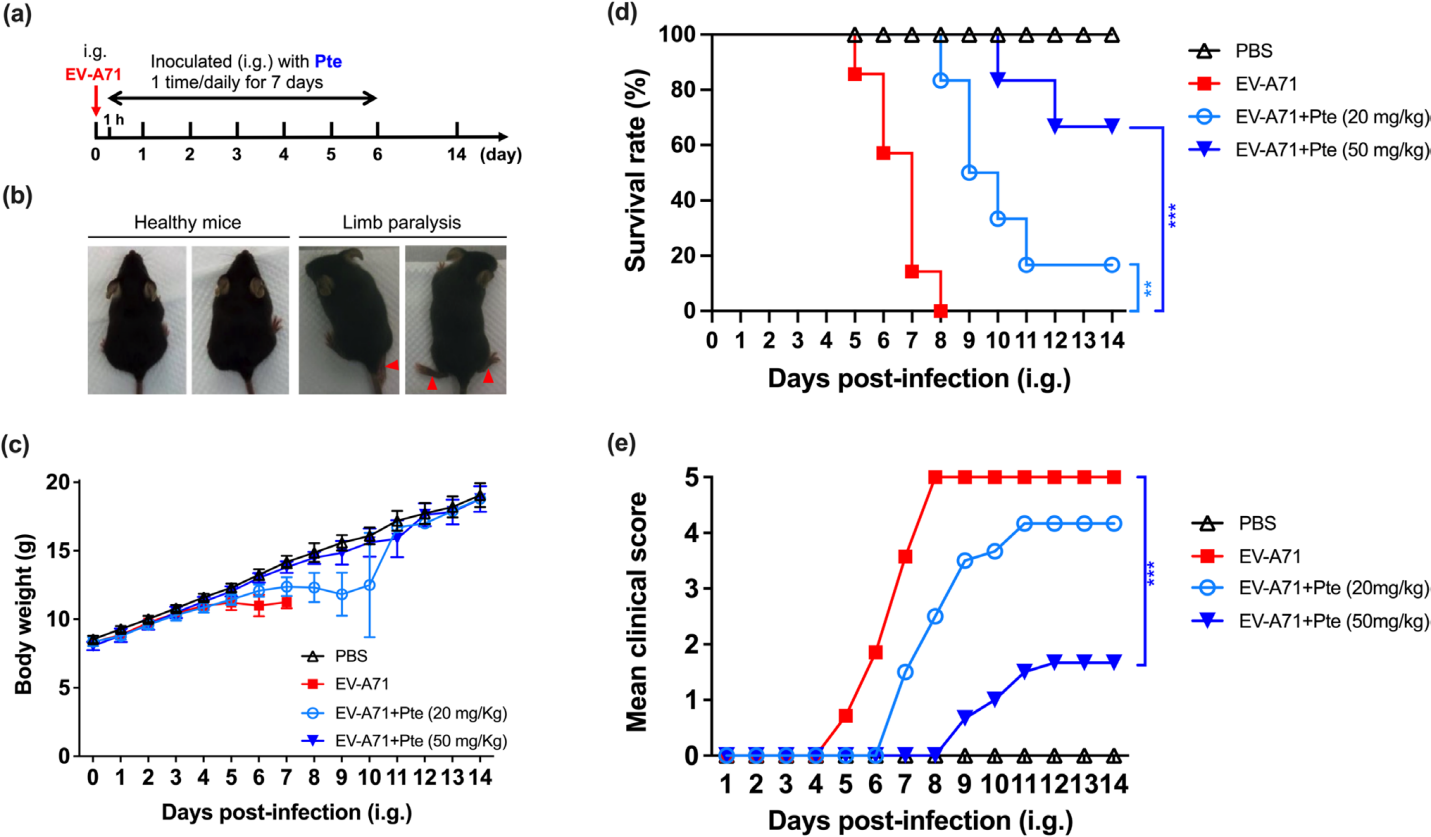
Oral administration of pterostilbene protects against EV‐A71 infection. (a) Schematic diagram of in vivo experiment. hSCARB2 mice (21‐d‐old) were intragrastrically (i.g.) infected with EV‐A71, and then inoculated (i.g.) once‐daily with 20 and 50 mg/kg Pte for 1 h after Day 0–6, consecutively for a total of 7 day. (b) Representative photographs of healthy mice in the EV‐A71 + Pte (50 mg/kg) group. In contrast, limb paralysis was observed among mice in the EV‐A71 alone group. (c) Body weight, (d) survival rate, and (e) clinical scores were recorded daily after infection for 14 day. Clinical scores were graded as: 0, healthy; 1, hair loss, wasting, or ruffled hair; 2, limb weakness; 3, paralysis in only one limb; 4, paralysis in 2–4 limbs; 5, death. *N* = 6–7. Data are expressed as the mean ± SEM. **: *p* < 0.01, ***: *p* < 0.001 versus the virus control group.

Based on the potent activity of Pte in the cell‐based assays, we next evaluated the efficacy of Pte in inhibiting EV‐A71 infection in a mouse model. hSCARB2 transgenic mice were i.g. infected with EV‐A71/MP4 (1 × 10^7^ PFU/mouse). After 1 h, mice were i.g. treated with PBS or Pte (20 and 50 mg/kg). Mice in all groups were treated with Pte once daily for a total of 7 days and the body weight, survival, and clinical scores of mice were monitored daily for 14 days (Figure 8 and Table S6). Figure 8b shows representative images of protected mice in the Pte (50 mg/kg)‐treated group with healthy limbs and in the untreated group (EV‐A71) with limb paralysis on day 6 post‐infection with EV‐A71. Mice treated with 50 mg/kg Pte maintained better body weight compared with those in the EV‐A71 and 20 mg/kg Pte groups (Figure 8c). We observed that hSCARB2 mice i.g. inoculated with EV‐A71 displayed ataxia and paralysis within 5–7 days post‐infection (dpi), with all animals dying by 8 dpi. However, compared with the untreated (EV‐A71) group, Pte treatment relieved symptoms of EV‐A71 infection and reduced mortality. Notably, the 20 mg/kg Pte‐treated mice presented severe front and rear limb paralysis at 7–9 dpi, with all paralyzed mice dying at 11 dpi. Increasing the dose to 50 mg/kg markedly improved the survival rate to approximately 70% at 14 dpi (*p* < 0.01) (Figure 8d) and notably alleviated the clinical signs of infection (*p* < 0.01) (Figure 8e). Overall, these results demonstrated the protective role of Pte against EV‐A71 infection in vivo.

## 4 Discussion

Recently, the National Enterovirus Surveillance System has revealed a substantial increase in the incidence of respiratory illnesses related to EV‐D68 in the United States following the COVID‐19 pandemic (Souverbielle et al. 2023). Additionally, the frequency of hand‐foot‐and‐mouth disease, primarily caused by EV‐A71 and coxsackievirus, has increased in Asia (Qiao et al. 2023). Taiwan reported a fatal case of EV‐D68 in 2023, and several enterovirus‐associated infant deaths have been documented in Europe (Grapin et al. 2023). Despite their global prevalence and the significant morbidity associated with enteroviral infections, specific antiviral therapies are limited. Therefore, developing broad‐spectrum antivirals against enteroviruses, including EV‐A71, EV‐D68, and coxsackievirus, is imperative.

In this study, we first demonstrated that Pte is a potent broad‐spectrum antiviral agent against EV‐A71 (C2, B4), EV‐D68 (B1, B3), CV‐A16, and CV‐B3. Furthermore, Pte demonstrated anti‐enteroviral activity superior to that of Res. The antiviral activities of Pte were attributed to its inhibitory effects on RNA expression and viral protein synthesis and its protective effects against CPE. Based on the results of time‐of‐addition and time‐of‐removal assays, we speculated that Pte significantly affects the early stages of the enterovirus life cycle. Based on the results of the attachment assay, we suggested that Pte may inhibit EV‐A71 and EV‐D68 entry by targeting either the viral particles or cellular receptor(s) of host cells. We demonstrated that Pte inactivated enteroviruses and inhibited EV‐A71 or EV‐D68 attachment to host cells by interfering with hSCARB2, hPSGL‐1, or hICAM‐5 binding. Furthermore, Pte stabilized EV‐A71 and EV‐D68 virions and limited their infectivity, possibly by restricting the dynamics of the capsid necessary for genome release. Molecular docking analysis showed that Pte prevented early infection by occupying a hydrophobic pocket near the canyon region of VP1, which is involved in pathogenesis, viral particle assembly, and cell entry (Wang, Hu, et al. 2022). By competitively binding and stably replacing pocket factors (sphingosine, C_10_–C_18_ fatty acids) in the hydrophobic pocket, Pte disrupted viral attachment to the specific receptor on host cells, thereby restricting the uncoating process required for genome release (Lin et al. 2018). This binding site has been identified as a potent inhibition site for WIN series compounds.

WIN series compounds, such as pleconaril, are synthetic antiviral agents developed to target enteroviruses and rhinoviruses (Bernard et al. 2015; Wald et al. 2019). Their mechanism of action involves binding to a hydrophobic pocket in the viral capsid (VP1) and preventing the uncoating process essential for viral RNA release and replication. Although pleconaril underwent clinical trials (Hayden et al. 2003), its widespread clinical use was limited owing to safety concerns, such as drug interactions, leading to its rejection by the FDA. Whereas Pte is a natural compound with multiple potential binding sites in enteroviral strains that can interfere with multiple stages of the viral life cycle. Furthermore, Pte not only interferes with the binding of EV‐A71 to hSCARB2 but also disrupts the binding of EV‐A71 to hPSGL‐1 and EV‐D68 to hICAM‐5. Other studies have suggested that hPSGL‐1 and hICAM‐5 may be associated with multiple regions on the surface of the enterovirus, which requires further investigation (Fang et al. 2021; Plevka et al. 2012). Given that both hSCARB2 and hICAM‐5 are highly expressed in the central nervous system (Sun et al. 2019), Pte may relieve neurotropism during enteroviral infection by restricting the access of viral binding sites to these receptors. These results indicated that Pte exhibited early preventive and therapeutic effects, mitigating severity before the worsening of infection, particularly by EV‐A71 and EV‐D68, which are associated with high‐risk neurological diseases, such as AFM.

The antiviral activity of Pte is related to its conjugated structure, which effectively regulates the fluctuations in virus‐induced oxidative stress (Montenegro‐Landívar et al. 2021). Moreover, the two major advantages of Pte are its higher stability and enhanced lipophilicity, which facilitate cellular accumulation and easier penetration of cell membranes and the blood–brain barrier (Lin, Leland, et al. 2020). Small molecules enable more effective targeting of lesions through systemic circulation, overcoming physiological barriers and inducing therapeutic effects (Liao et al. 2019). Overall, these factors contribute to the superior bioactivity of Pte against multiple enteroviral strains relative to that of Res. Additionally, Pte exerts antiviral effects against other viruses, including SARS‐CoV‐2 (Ter Ellen et al. 2021), HIV‐1 (Chan et al. 2017), influenza virus (Zhang et al. 2024), and human cytomegalovirus (Wang, Zhou, et al. 2022).

Furthermore, we performed molecular docking analyses and multiple sequence alignments of the VP1 protein from EV‐A71, EV‐D68, CV‐A16, and CV‐B3 to evaluate the conservation of the Pte binding sites across these strains. The molecular docking results indicated that Pte interacts with specific residues in the hydrophobic pocket of VP1 and with a binding mode similar to that of known capsid‐binding inhibitors in the VP1 pocket of the four enteroviruses (Figure S1). Furthermore, multiple sequence alignments revealed that Pte‐binding residues are highly conserved among the tested enterovirus strains (Figure 7). The results of the VP1 amino acid sequence alignment of EV‐A71 from various genotypes showed that the Pte‐binding residues are fully conserved (Table S2). Tables S3 **–** S5 show that most of the key interacting residues are highly conserved in EV‐D68, CV‐A16, and CV‐B3, suggesting the broad‐spectrum activity of Pte against enteroviruses. Additionally, the results suggest that Pte targets conserved sites within the VP1 pocket that are critical for viral stability and uncoating (Liu, Sheng, Fokine, et al. 2015; Plevka et al. 2013). Moreover, the binding mechanism of Pte is similar to that of known capsid‐binding inhibitors (such as pleconaril), which also target conserved hydrophobic pockets across enterovirus species (Table S1). As summarized in Table S1, Pte interacts with highly similar residues within VP1 as WIN51711, pleconaril, and GPP3, further supporting its potential as a broad‐spectrum antiviral compound.

Overall, our studies showed that the Pte‐binding residues within the hydrophobic pocket of VP1 are highly conserved among multiple enteroviruses, suggesting a potential for broad‐spectrum inhibition. Furthermore, although the residues variation in the Pte binding site is small, the effects of residue size and structural variation on the VP1 pocket also need to be considered. Even minor amino acid substitutions may alter the size of the hydrophobic pocket, potentially making it narrower and less accessible to inhibitors, thereby reducing the ability to interact effectively. Studies have shown that the replacement of a single amino acid can significantly affect the efficacy of a drug (Ho et al. 2016; Shih et al. 2004). In the future, further experimental validation is required to confirm the antiviral activity of Pte against diverse clinical strains from different outbreaks and natural variants of enteroviruses. The additional studies will be critical to confirm the potential of Pte as an antiviral agent and to evaluate its efficacy against different enterovirus strains.

To date, the effectiveness of Pte against enteroviruses has not been verified in animal experiments. Neonatal and immunodeficient mice are typically used for in vivo experiments (Liu et al. 2020; Liou et al. 2016); however, viral susceptibility declines in mice over a few weeks of age while the use of immunodeficient mice can obscure the role of the innate immune system in EV‐A71 infection. To investigate the antiviral activity of Pte in vivo, hSCARB2 transgenic mice (21‐d‐old) were intragastrically inoculated with a murine‐adapted EV‐A71/MP4 strain (Lin et al. 2021). hSCARB2 expression enhanced the binding of EV‐A71 to the cell surface, increasing susceptibility to infection and resulting in higher infection efficiency than that mediated by hPSGL‐1 (Liu et al. 2012; Yamayoshi et al. 2013). After infection, these mice exhibited symptoms of paralysis, similar to those observed in fatal human cases. Successful infection was determined by the onset of neurological symptoms, including limb weakness, paralysis, and mortality (Figure 8b,d,e). Importantly, 21‐d‐old mice are equivalent to 1–2‐year‐old humans, which is consistent with the period when humans are most susceptible to infection. Therefore, our transgenic mouse model was appropriate for infectivity and therapeutic research as it closely mimics the pathological characteristics in humans (Yang et al. 2019). Moreover, oral‐gavage‐based inoculation for gastrointestinal mucosal infections more closely mimics the natural routes of enteroviral infection in human infants than subcutaneous, intraperitoneal, or intracerebral injections (Yang et al. 2019). Additionally, administering oral medications to 21‐d‐old mice offers advantages over neonatal mice due to their more developed digestive system, which allows for more efficient drug absorption. Their larger size facilitates more precise dosing and minimizes the risk, leading to improved reliability in experimental results. A recent study demonstrated that Pte enhanced innate immune activation during enteroviral infection (Yang et al. 2023). These results emphasized the importance of Pte for the immune system (Wu et al. 2023; Yue et al. 2017). We speculated that Pte may not only possess anti‐enteroviral activity but may also be able to inhibit enteroviral infection by stimulating innate immunity (Lin, Leland, et al. 2020). In our in vivo study, Pte at 50 mg/kg significantly increased survival rates to approximately 70%, compared to the control group (0% survival). Pte‐treated mice also exhibited a significant reduction in clinical scores, indicative of improved disease outcomes. This protective effect is comparable to the 80% survival rate observed with pleconaril (80 mg/kg) in previous EV‐A71 mouse models (Zhang et al. 2012). In contrast, ribavirin provided no protection, with all treated mice succumbing to the infection, regardless of the dose administered (80, 20, 5, 1.25, and 0.375 mg/kg) (Zhang et al. 2012). These findings suggest that the efficacy of Pte is comparable to pleconaril and superior to ribavirin in treating enterovirus infections, warranting further evaluation in translational studies.

Furthermore, the selection of the doses (20 and 50 mg/kg/day) was based on previous pharmacological studies, established human‐to‐mouse dose conversion principles, and supporting literature on Pte usage in animal models. A pharmacological study indicated that oral administration of high doses of Pte (300 or 3000 mg/kg/d) for 28 days did not induce organ‐specific toxicity and mortality in mice (Lin, Shih, et al. 2020; Ruiz et al. 2009). Based on the formula human equivalent dose (mg/kg) = animal dose (mg/kg) × (animal Km/human Km), (human Km = 37, mouse Km = 3) (Nair and Jacob 2016), a mouse dose of 50 mg/kg equals a human dose of 4.05 mg/kg, that is, 243 mg Pte for a human weighing 60 kg. Human clinical trials have confirmed that a daily intake of up to 250 mg of Pte is safe in humans (Lin, Leland, et al. 2020; Riche et al. 2013). In this study, the daily dosage for humans was approximately 243 mg of Pte, which is still considered safe. Previous studies have demonstrated the efficacy of Pte in various disease models at comparable dosages to the one used in our study. For example, intragastric administration of Pte at doses of 30 or 60 mg/kg/day improved the performance of mice with influenza A virus‐induced acute lung injury (Zhang et al. 2024). Additionally, oral administration of Pte (100 mg/kg body weight) once daily for 14 days effectively reduced inflammation in a colonic inflammation model (Yashiro et al. 2020). Several studies on stilbenoids, including Pte, showed that effective doses ranged from 10 to 100 mg/kg/day, depending on the disease model (Koh et al. 2021). Accordingly, we selected doses of 20 and 50 mg/kg/day, which fall within the effective range reported in the literature, ensuring a balance between efficacy and safety. Overall, our dosage selection is strongly supported by toxicological safety data, established dose conversion principles, and previous animal studies, making it appropriate for evaluating the antiviral efficacy of Pte. Future studies could also include Pte‐loaded nanoparticles to achieve a reduction in cytotoxicity and an increase in selectivity index. Therefore, Pte has the potential to be used as a safe and effective anti‐enteroviral drug in the future. However, this study is limited by the absence of a positive control. Because no FDA‐approved antivirals exist for enteroviruses, a standard positive control was unavailable. Future studies could compare Pte with experimental VP1‐targeting inhibitors such as pleconaril and vapendavir.

Overall, this study demonstrated that Pte exhibits antiviral activity against multiple viral strains, including EV‐A71, EV‐D68, coxsackievirus A16, and coxsackievirus B3. Specifically, Pte exerts its antiviral effects by binding to the hydrophobic pocket of the VP1 protein, preventing the conformational changes necessary for viral uncoating and RNA release. This action stabilizes the viral capsid and inhibits early stages of viral replication. Additionally, Pte inactivates viral particles and blocks their attachment to host cells by disrupting virus‐receptor interactions, ultimately preventing EV‐A71‐induced neurological symptoms in vivo. Additionally, our sequence alignment of VP1 highlights the high conservation of Pte‐binding residues across multiple enteroviruses. Collectively, these findings suggest that Pte may serve as a broad‐spectrum antiviral agent for the prevention and treatment of large‐scale enteroviral infections.

## Author Contributions

**Kuan‐Ting Chuang:** data curation, formal analysis, investigation, methodology, software, validation, writing – original draft. **Siao‐Cian Pan:** data curation, investigation, validation, writing – original draft. **Bor‐Luen Chiang:** conceptualization, project administration, supervision, writing – review and editing. **Shih‐Hsun Chen:** software, visualization. **Min‐Hsiung Pan:** resources, supervision. **Yu‐Li Chen:** formal analysis, software. **Cheng‐Sheng Lin:** data curation, formal analysis, methodology. **Chun‐Kai Pan:** investigation, validation. **Jing‐Yi Lin:** methodology, validation. **Yu‐Li Lin:** conceptualization, funding acquisition, project administration, resources, supervision, writing – review and editing.

## Conflicts of Interest

The authors declare no conflicts of interest.

## Supporting information


**Data S1.** Supporting Information.

## Data Availability

The data that support the findings of this study are available from the corresponding author upon reasonable request.
