# Supplementary material for: Pterostilbene Exhibits Broad‐Spectrum Antiviral Activity by Targeting the Enterovirus Capsid, Inactivating Viral Particles, Blocking Viral Binding, and Protecting Mice From Lethal EV‐A71 Challenge
Source: Phytother Res. 2025 Apr 16;39(6):2672–88. doi: 10.1002/ptr.8496 (PMC12178775; doi:10.1002/ptr.8496)
Supplement: Supplementary file 1 — Data S1. Supporting Information. [file PTR-39-2672-s001.docx]

**Supporting information**

**Pterostilbene Exhibits Broad-Spectrum Antiviral Activity by Targeting the Enterovirus Capsid, Inactivating Viral Particles, Blocking Viral Binding, and Protecting Mice from Lethal EV-A71 Challenge**

**Short title:** Pterostilbene Against Enterovirus Infections

Kuan-Ting Chuang^a^, Siao-Cian Pan^a^, Bor-Luen Chiang^a,b,c^, Shih-Hsun Chen^d^, Min-Hsiung Pan^e^, Yu-Li Chen^f^, Cheng-Sheng Lin^a^, Chun-Kai Pan^a^, Jing-Yi Lin^g^, and Yu-Li Lin^a,^*

^a^Department of Medical Research, National Taiwan University Hospital, Taipei, Taiwan

^b^Department of Pediatrics, National Taiwan University Hospital, Taipei, Taiwan

^c^Graduate Institute of Immunology, College of Medicine, Taipei, Taiwan

^d^Institute of Biochemical Sciences, National Taiwan University, Taipei, Taiwan

^e^Institute of Food Science and Technology, National Taiwan University, Taipei, Taiwan

^f^Research Center for Chinese Herbal Medicine, Graduate Institute of Health Industry Technology, College of Human Ecology, Chang Gung University of Science and Technology, Taoyuan, Taiwan

^g^Department of Clinical Laboratory Sciences and Medical Biotechnology, College of Medicine, National Taiwan University, Taipei, Taiwan

*Corresponding author

Yu-Li Lin, Department of Medical Research, National Taiwan University Hospital, 10F, No. 7, Zhongshan S. Rd., Zhongzheng Dist., Taipei City 100225, Taiwan (R.O.C.)

Tel: (886) 2-2312-3456 ext. 263170; Fax: (886) 2-2322-5329

E-mail address: [linyuli888@gmail.com](mailto:linyuli888@gmail.com) (Yu-Li Lin)

**
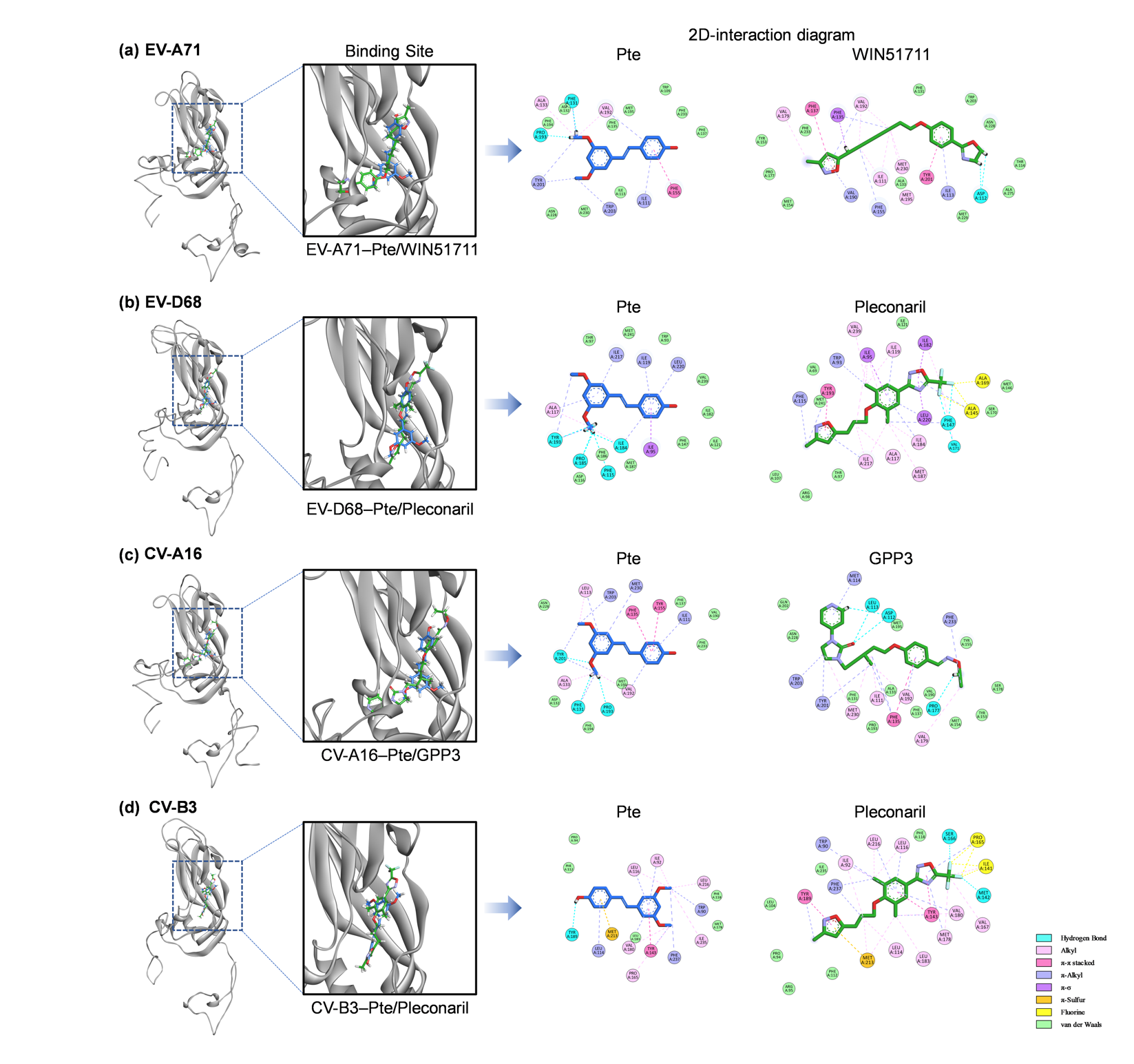
**

**Figure S1.** Molecular docking analysis of pterostilbene (Pte) and reference inhibitors within the VP1 hydrophobic pocket of four enteroviruses. (a) EV-A71 (PDB code: 3ZFF) binding site with Pte and WIN51711; (b) EV-D68 (7TAG) binding site with Pte and pleconaril; (c) CV-A16 (5ABJ) binding site with Pte and GPP3; and (d) CV-B3 (1COV) binding site with Pte and pleconaril. The left panels show the full viral capsid structure with a magnified view of the VP1 binding pocket. The middle panels compare the docked Pte ligand with known inhibitors. The right panels present the 2D-interaction diagrams, highlighting amino acid residues interacting with Pte (blue) and reference inhibitors (green). Oxygen, nitrogen, and fluorine atoms are depicted in red, purple, and cyan, respectively.

Figure S1 illustrates the molecular docking interactions of pterostilbene (Pte) and known VP1 inhibitors (WIN51711, pleconaril, and GPP3) (Figure S2) within the VP1 hydrophobic pocket of four enteroviruses (EV-A71, EV-D68, CV-A16, and CV-B3), which were simulated using BIOVIA Discovery Studio 2024 (Dassault Systems, San Diego, CA, USA). The left panels in Figure S1 depict the 3D structural binding sites, highlighting the spatial positioning of Pte and the reference inhibitors. The magnified views of the binding sites show the interactions of each compound within the VP1 pocket, which are followed by 2D-interaction diagrams that map the key residues involved in ligand binding. These diagrams reveal a network of hydrogen bonds, π–π stacking, and hydrophobic interactions contributing to the ligand stability within the pocket. The interacting residues of Pte highly structurally overlapped with the reference inhibitors, suggesting that Pte stabilizes the VP1 pocket similarly to established capsid-binding antivirals. These findings provide structural insights into the potential broad-spectrum antiviral activity of Pte, supporting the role of Pte in enterovirus inhibition through preventing uncoating and RNA release.

**Figure S2.** Chemical structures of (a) pterostilbene, (b) WIN51711, (c) pleconaril, and (d) 3-(4-pyridyl)-2-imidazolidinone (GPP3).

**Table S1.** Comparison of interacting residues between pterostilbene and known capsid-binding inhibitors (WIN51711, pleconaril, and GPP3) within the VP1 hydrophobic pocket of four enteroviruses (EV-A71, EV-D68, CV-A16, and CV-B3).

Colored amino acids highlights indicate identical interacting residues between pterostilbene and known capsid-binding inhibitors (WIN51711, pleconaril, and GPP3), showing similar binding profiles.

The results of the analysis of the residues interacting within the VP1 hydrophobic pocket of four enteroviruses (EV-A71, EV-D68, CV-A16, and CV-B3) revealed that Pte contains key binding sites for known capsid-binding inhibitors, including WIN 51711, pleconaril, and GPP3 (Table S1). These results indicate that Pte interacts with the conserved amino acids within the VP1 pocket, which are also the targets of these antiviral compounds. The colored residues in Table S1 highlight the the interacting residues that are identical within the hydrophobic pocket between Pte and the known VP1-target inhibitors, indicating similar binding properties. These findings suggested that Pte, similar to its positive control counterparts, stabilizes the viral capsid via interacting with conserved structural elements, potentially limiting viral uncoating and RNA release, a mechanism contributing to its broad-spectrum antiviral activity against enteroviruses.

**Table S2.** Amino acid sequence alignment of the EV-A71 VP1 protein of various genotypes.

Dots (•) indicate identical amino acids among the different genotypes relative to EV-A71 B3.

Table S2 provides the amino acid sequence alignment of the EV-A71 VP1 protein across various genotypes, specifically focusing on the residues involved in Pte binding. The reference strain EV-A71 B3 is listed at the top of Table S2 with its VP1 residues. Dots (•) indicate residues that are identical in the other EV-A71 genotypes to those in the B3 strain. The conservation of these residues across all tested genotypes suggests that the binding sites of Pte in the VP1 hydrophobic pocket are highly conserved among different EV-A71 strains, implying that Pte may exhibit broad-spectrum antiviral activity against diverse EV-A71 genotypes. These findings demonstrated the potential universal applicability of Pte as a VP1-targeting inhibitor.

**Table S3.** Amino acid sequence alignment of the EV-D68 VP1 protein of various genotypes.

Dots (•) indicate identical amino acids among the different genotypes relative to EV-D68 US/MO/14-18947.

Table S3 shows the amino acid sequence alignment of the EV-D68 VP1 protein across multiple strains, with EV-D68 US/MO/14-18947 as the reference strain. The chemical properties of Ile and Met were similar, except for the Met substitution at position 182, suggesting that most of the Pte-interacting residues are highly conserved. This high conservation degree suggests that the VP1 binding sites of Pte in EV-D68 are structurally stable across different genotypes; therefore, Pte may exhibit broad-spectrum antiviral activity against diverse EV-D68 genotypes.

**Table S4.** Amino acid sequence alignment of the Coxsackievirus A VP1 protein of various genotypes.

Dots (•) indicate identical amino acids among the different genotypes relative to Coxsackievirus A16.

Table S4 provides the amino acid sequence alignment of the VP1 protein across various Coxsackievirus A genotypes, using Coxsackievirus A16 as the reference strain. Variations were observed at positions 113, 155, 192, 195, and 230 among the aligned strains. The chemical properties at positions 113, 155, 192, 195, and 230 were highly similar, whereas those at position 132 was weak similarity. However, the most binding residues are highly conserved.

**Table S5.** Amino acid sequence alignment of the Coxsackievirus B VP1 protein of various genotypes.

Dots (•) indicate identical amino acids among the different genotypes relative to Coxsackievirus B3.

Table S5 presents the amino acid sequence alignment of the Coxsackievirus B VP1 protein across multiple strains, using Coxsackievirus B3 as the reference strain. Variations were observed at positions 92, 94, 112, 114, 180, 183, and 216 among the aligned strains. The chemical properties at positions 92, 114, 180, 183, and 216 were highly similar, whereas those at position 94 and 112 were dissimilar. However, the most binding residues remained highly conserved.

**Table S6.** Effects of pterostilbene on clinical score changes during EV-A71 infection in hSCARB-2 Tg mice.

**Table S7.** The source, catalog number, and lot of antibodies and recombinant proteins.
